# BALB/c mice immunized with a combination of virus-like particles incorporating Kaposi sarcoma-associated herpesvirus (KSHV) envelope glycoproteins gpK8.1, gB, and gH/gL induced comparable serum neutralizing antibody activity to UV-inactivated KSHV

**DOI:** 10.18632/oncotarget.15605

**Published:** 2017-02-22

**Authors:** Anne K Barasa, Peng Ye, Meredith Phelps, Ganapathiram T Arivudainambi, Timelia Tison, Javier Gordon Ogembo

**Affiliations:** ^1^ Department of Experimental Therapeutics, Beckman Research Institute of City of Hope, Duarte, CA, USA; ^2^ Department of Medicine, University of Massachusetts Medical School, Worcester, MA, USA; ^3^ Department of Human Pathology, University of Nairobi, Nairobi, Kenya

**Keywords:** kaposi sarcoma-associated herpes virus, glycoproteins, virus-like particles, cancer, prophylactic vaccine

## Abstract

Infection with Kaposi sarcoma-associated herpesvirus (KSHV) is estimated to account for over 44,000 new cases of Kaposi sarcoma annually, with 84% occurring in Africa, where the virus is endemic. To date, there is no prophylactic vaccine against KSHV. KSHV gpK8.1, gB, and gH/gL glycoproteins, implicated in the virus entry into host cells, are attractive vaccine targets for eliciting potent neutralizing antibodies (nAbs) against virus infection. We incorporated gpK8.1, gB, or gH/gL on the surface of virus-like particles (VLPs) and characterized these VLPs for their composition, size, and functionality. To determine which viral glycoprotein(s) elicit the most effective serum-nAbs, we immunized BALB/c mice with gpK8.1, gB, or gH/gL VLPs individually or in combination. Neutralizing antibody assay revealed that sera from mice immunized with the VLPs inhibited KSHV infection of HEK-293 cells in a dose-dependent manner. As a single immunogen, gpK8.1 VLPs stimulated comparable nAb activity to that of UV-inactivated KSHV (UV-KSHV). In contrast, UV-KSHV stimulated higher titers of nAb compared to gB (p = 0.0316) or gH/gL (p = 0.0486). Mice immunized with the combination of gB and gH/gL VLPs had a better nAb response than those immunized with either gB (p = 0.0268), or gH/gL (p = 0.0397) as single VLP immunogens. Immunization with any VLP combination stimulated comparable nAb activity to UV-KSHV serum. Our data provide the first evidence that KSHV gpK8.1, gB, and gH/gL glycoproteins can be incorporated onto the surface of VLPs and used as prophylactic vaccine candidates, with potential to prevent KSHV infection.

## INTRODUCTION

Kaposi sarcoma-associated herpesvirus (KSHV), also known as human herpesvirus-8 (HHV-8), is the etiologic agent of Kaposi sarcoma (KS), an endothelial cell tumor [1]. KSHV is also associated with primary effusion lymphoma and multicentric Castleman disease lymphoproliferative disorders [2]. KSHV transmission occurs mainly via saliva exchange, sexual contact, and rarely, through organ transplantation and blood transfusion [3–9]. The seroprevalence of KSHV varies globally, with high rates in parts of Africa and South America (30-60%, considered to be endemic), intermediate rates in the Mediterranean region (4-35%), and low rates in North America, Western Europe, and Asia (<10%) [10–13]. KS is most frequent in regions with high KSHV seroprevalence, such as African and Mediterranean countries [14].

KSHV-associated malignancies predominantly present in people with primary and acquired immunodeficiencies, including acquired immunodeficiency syndrome (AIDS) and iatrogenic immunosuppression in organ transplant recipients [15, 16]. KS is one of the most frequent malignancies seen in AIDS patients [17, 18]. It is a major cause of morbidity and mortality in adults in sub-Saharan Africa, where the prevalence of HIV/AIDS is high, and it is an emerging problem for children [19–21]. Despite a significant reduction in the incidence of KS since the introduction of highly active antiretroviral therapy (HAART), there are still approximately 44,000 new cases of KS worldwide each year, with 84% occurring in Africa [22]. Furthermore, only 50% of patients achieve complete resolution of KS lesions with HAART [23]. Thus, there is a need for improved preventive and therapeutic approaches to KS.

Prophylactic vaccines typically stimulate an individual's humoral immune system to produce neutralizing antibodies against the surface glycoprotein(s) mediating the infectious agent's entry [24, 25]. The role of neutralizing antibodies in preventing KSHV infection or KSHV-associated malignancies has not been determined. To date, efforts to develop a prophylactic vaccine against KSHV have been lacking, with no preclinical or clinical vaccine trial reported in the past three decades, despite the AIDS pandemic [26]. A KSHV vaccine would be highly beneficial for at-risk populations, e.g., those at high risk of HIV infection, people living in KSHV-endemic areas, or individuals undergoing iatrogenic immunosuppression after organ or stem cell transplantation. An effective KSHV vaccine could even eradicate the virus and its associated malignancies in areas where low seroprevalence rates are reported.

Like all other herpesviruses, KSHV enters target cells through a multi-step process that involves interactions between multiple viral envelope glycoproteins and host cell surface molecules that include attachment and fusion receptors [27, 28]. The KSHV envelope glycoproteins implicated in virus-cell attachment and fusion, and which facilitate viral entry are gpK8.1, gB, and gH/gL. Both gpK8.1 and gB are thought to initiate viral entry by binding to host cell surface heparan sulfate [29, 30]. This leads to conformational changes that allow access to specific entry receptors, including integrins and ephrin receptors, which facilitate viral uptake into the host cells [31]. gB binds to integrin receptors, while gH and gL form a non-covalently linked complex that interacts with the ephrin receptor EphA2 [32]. Studies have shown that the gH/gL complex is indispensable for viral entry, and that antibody blockade of gH/gL affects KSHV target cell entry without affecting binding [33].

We developed KSHV envelope glycoprotein-based virus-like particles (VLPs) as prophylactic vaccine candidates. VLPs are self-assembling structures composed of one or more viral proteins. They can serve as delivery systems to present antigens from different types of pathogens [34]. The size and structure of VLPs closely resembles those of native virus, but they are non-infectious, as they lack viral genetic material. These qualities make VLPs a highly immunogenic and safe vaccine platform for oncogenic viruses such as KSHV [35].

This study demonstrates that targeting viral entry as a strategy for prevention of KSHV infection and its associated malignancies is feasible. We used the Newcastle disease virus (NDV) platform to generate VLPs bearing KSHV gpK8.1, gB, or gH/gL envelope glycoproteins. We immunized wild-type BALB/c mice and measured neutralizing antibody response. Our results confirm that KSHV glycoprotein-based VLPs are immunogenic and generate a KSHV-specific immunoglobulin G (IgG) antibody response in immunized mice. In addition, *in vitro* neutralization assays performed using recombinant KSHV tagged with enhanced green fluorescent protein (KSHV-eGFP) showed that antibodies generated by KSHV glycoprotein-based VLP-immunized mice can inhibit KSHV infection *in vitro*.

## RESULTS

### Construction of chimeric gpK8.1-F, gB-F, gH-F, and gL-HN cDNA plasmids, and purification and characterization of KSHV VLPs

**Figure 1:**
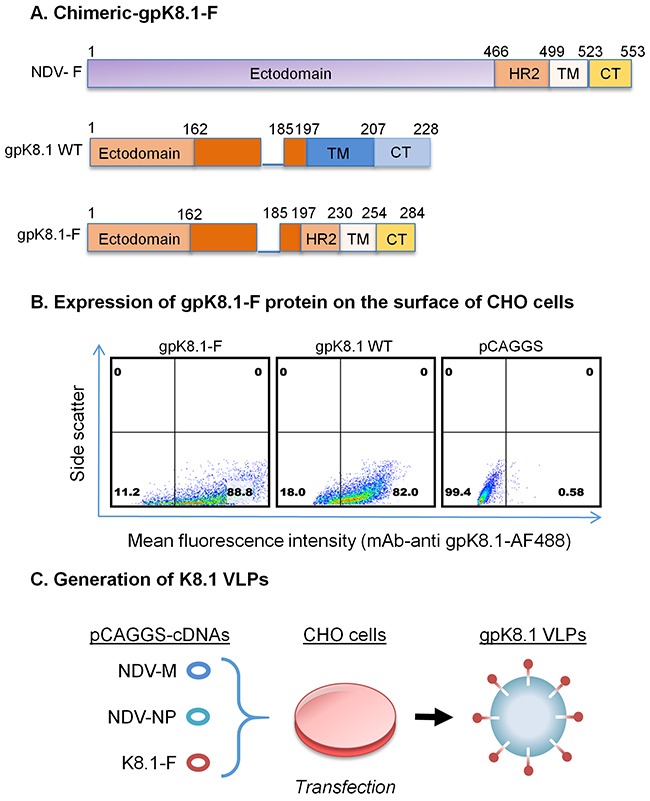
Schematic representation of the construction of chimeric KSHV gpK8.1-F and production of gpK8.1 VLPs **A**. gpK8.1-F plasmid constructs (not to scale) showing full-length NDV-F (top), full-length/wild-type (WT) gpK8.1 (middle), and chimeric gpK8.1-F (bottom). **B**. Flow cytometric analysis for surface expression of gpK8.1 protein on 10^6^ CHO cells transfected with 1 μg of pCAGGS gpK8.1-F chimera, pCAGGS gpK8.1 WT (positive control), and empty pCAGGS vector (negative control) was performed at 48 h post-transfection. Transfected cells were resuspended in PBS, stained with anti-gpK8.1 mAb, which detects the ectodomain of the protein, followed by secondary antibody goat-anti-mouse IgG conjugated to AF488. From left, gpK8.1-F chimeric protein is robustly expressed on the surface of CHO cells, similar to WT gpK8.1 (middle panel), and no gpK8.1 protein was detected in CHO cells transfected with pCAGGS alone (negative control). **C**. Illustration of co-transfection of CHO cells with three cDNA plasmids required to make VLPs, pCAGGS cDNAs NDV-M, NDV-NP and gpK8.1-F, resulting in release of fully-assembled gpK8.1 VLPs. cDNAs NDV-M and NDV-NP have been described (see Materials and Methods).

To generate KSHV gpK8.1 VLPs, we first designed and synthesized a chimeric KSHV gpK8.1-F construct by fusing the KSHV gpK8.1 ectodomain to the NDV fusion protein (F) heptad repeat 2 (HR2), transmembrane (TM), and cytoplasmic (CT) domains (Figure 1A). The construct was cloned into a mammalian expression vector (pCAGGS) and the fidelity of the chimeric gene was verified by sequencing.

To demonstrate that chimeric gpK8.1-F is functionally expressed on the surface of cells, we transfected Chinese hamster ovary (CHO) cells with the gpK8.1-F plasmid or relevant controls. Forty-eight hours post-transfection, cells were stained with anti-gpK8.1 mouse monoclonal antibody (mAb), which detects the ectodomain of the protein, followed by secondary anti-mouse antibody conjugated to Alexa Fluor (AF488) fluorochrome. Flow cytometry showed that CHO cells transfected with the pCAGGS-gpK8.1-F chimera robustly expressed gpK8.1 (88% expression), similar to cells transfected with pCAGGS-gpK8.1 full-length (WT) (82% expression), which served as a positive control (Figure 1B). Cells transfected with empty pCAGGS vector (negative control) did not express gpK8.1.

To assemble and produce gpK8.1 VLPs, equal amounts of gpK8.1-F, NDV-matrix (M), and NDV-nucleocapsid protein (NP) cDNA plasmids were co-transfected into CHO cells (Figure 1C). The cell supernatant containing VLPs was collected every 24h until 96 h post-transfection. VLPs were purified as previously described [25, 36].

**Figure 2:**
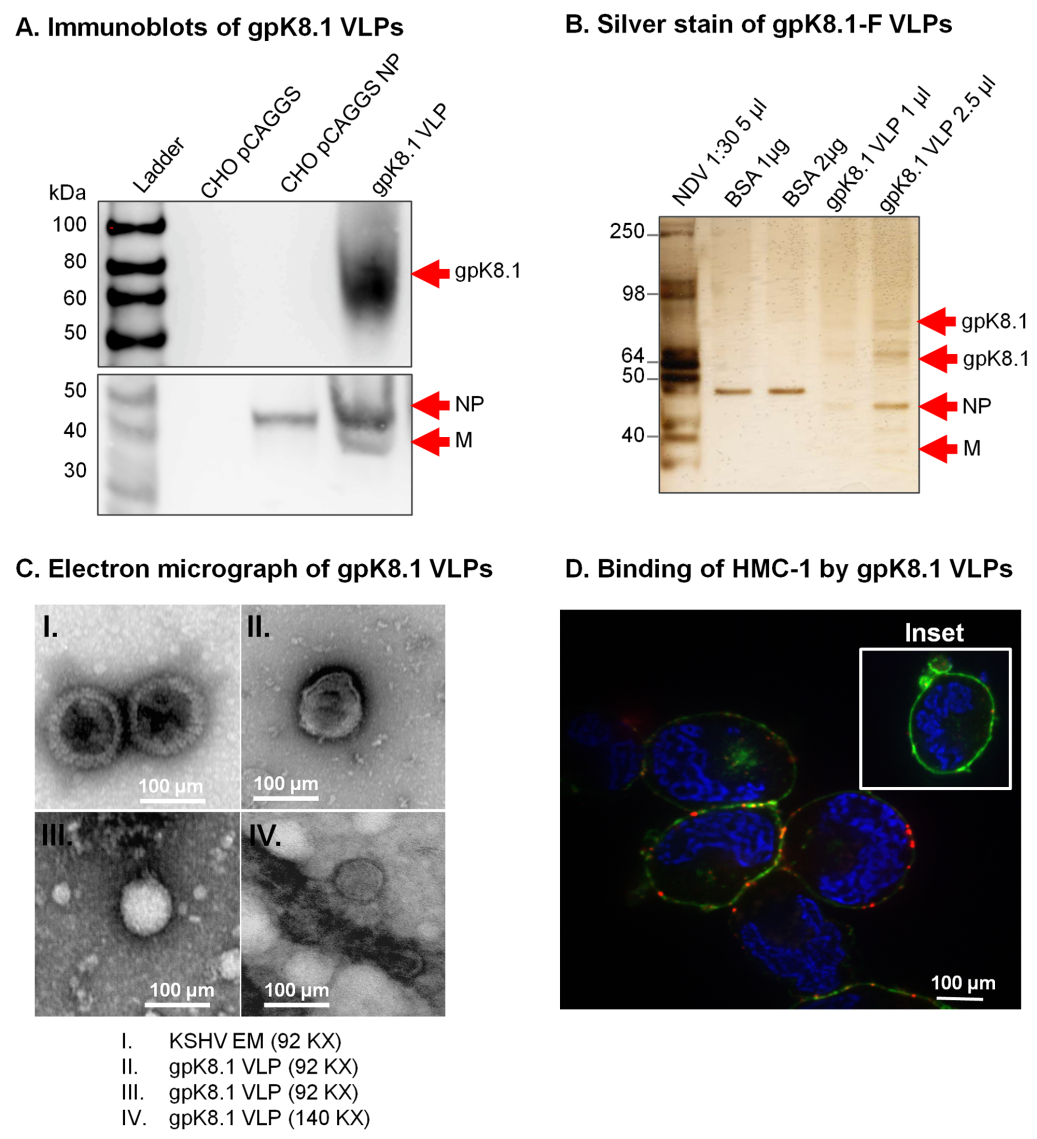
Characterization of KSHV gpK8.1 VLPs **A**. CHO cells transfected with empty pCAGGS or pCAGGS-NP, or purified gpK8.1 VLPs were lysed and analyzed by immunoblot using anti-gpK8.1 mAb and rabbit polyclonal anti-NDV to detect various VLP components. Anti-gpK8.1 (top panel) detected gpK8.1 (62-72 KDa) in VLP lysate (lane 4), but not in CHO pCAGGS or CHO NP (negative controls, lanes 2-3). Anti-NDV (bottom panel; a gift of Dr. T. Morrison, University of Massachusetts Medical School) detected NP alone in lysate from CHO NP (lane 3), and NP and M in gpK8.1 VLPs (lane 4), but not in CHO pCAGGS lysate (negative control, lane 2). **B**. Silver stain was used to visualize VLP purity relative to purified NDV. Arrows indicate viral/VLP components in 5 μl purified NDV diluted 1:30 (lane 1), and 1 μl and 2.5 μl of purified gpK8.1 VLPs diluted 1:40 (lanes 4-5). Lanes 2 and 3 were loaded with 1 μg and 2 μg BSA, respectively for protein quantification. **C**. Electron microscopy showing structural similarity between KSHV virions and gpK8.1 VLPs. Purified VLPs were dialyzed against 1 L TNE buffer to remove residual sucrose, incubated with 3% bovine serum albumin (BSA) in TNE for 45 min, and embedded on a grid. 5 μl of the virus/VLP at 1:40 dilution was individually added to the grid for 1 h at room temperature. After two final washes, the grids were negatively stained with 12% phosphotungstic acid (pH 7) for 15 sec, air dried for 30 min, and examined using a Tecnai transmission electron microscope (FEI). **D**. Confocal microscopy shows gpK8.1 VLPs binding to lipid rafts on HMC-1 cells. Purified gpK8.1 VLPs were incubated with HMC-1 cells for 10 min at room temperature and stained with anti-gpK8.1, followed by secondary antibody conjugated with Alexa-Fluor 594 (red) to detect gpK8.1 VLPs, cholera toxin (green) to detect lipid rafts, and DAPI (blue) to detect HMC-1 nuclei. The red and green staining shows VLPs bound cell membranes; HMC-1 cells not incubated with VLPs did not stain red (negative control, inset). VLPs (red) binding to lipid rafts are not seen in HMC-1 cells not incubated with VLPs (negative control, inset).

To confirm that gpK8.1, NP, and M were incorporated into gpK8.1 VLPs, we lysed purified VLPs in RIPA buffer and analyzed the lysates for protein composition by immunoblot (Figure 2A). As expected, anti-gpK8.1 mAb detected gpK8.1 (˜60–72 kDa) in purified VLPs (top panel, lane 4). No gpK8.1 protein was detected in lysates from CHO cells transfected with either empty pCAGGS vector or pCAGGS-NP (negative controls). Detection with rabbit polyclonal anti-NDV recognized NDV-NP in cells transfected with pCAGGS-NP and both NDV-NP and -M components in purified VLPs (bottom panel, lanes 3-4). No NDV components were detected in CHO cells transfected with empty pCAGGS vector (negative control). Immunoblot of gpK8.1 VLPs relative to additional controls (irrelevant Epstein-Barr VLPs, purified NDV, lytically induced iSLK.219 KSHV-eGFP-expressing cells, purified Epstein-Barr virus, and iSLK cells) confirmed that KSHV VLPs contained only the expected components (Supplementary Figure 1).

To assess the purity of gpK8.1 VLPs, we used silver stain; NDV lysate served as a positive control, and BSA was used for quantification of proteins. Bands of predicted sizes were detected for gpK8.1 (60-72 kDa), NDV-NP (˜50 kDa), and NDV-M (35-45 kDa) (Figure 2B). Electron microscopy of gpK8.1 VLPs confirmed that gpK8.1 VLPs structurally resembled KSHV (Figure 2C).

To confirm that chimeric gpK8.1-F proteins incorporated into VLPs were in the right conformation and able to bind cellular surface receptors, purified gpK8.1 VLPs were incubated with human mast cells 1 (HMC-1) susceptible to KSHV infection. Cells incubated with VLPs were stained with anti-gpK8.1 mAb conjugated with AF-594 (red) to detect gpK8.1 VLPs, cholera toxin (green) to detect lipid rafts, and DAPI (blue) to detect HMC-1 nuclei (Figure 2D). HMC-1 cells not incubated with VLPs were used as negative control (inset). Stained cells were analyzed by confocal microscopy as described in Materials and Methods, and showed binding of VLPs to the lipid rafts in the cell membranes of VLP-incubated cells, indicative of functional binding.

**Figure 3:**
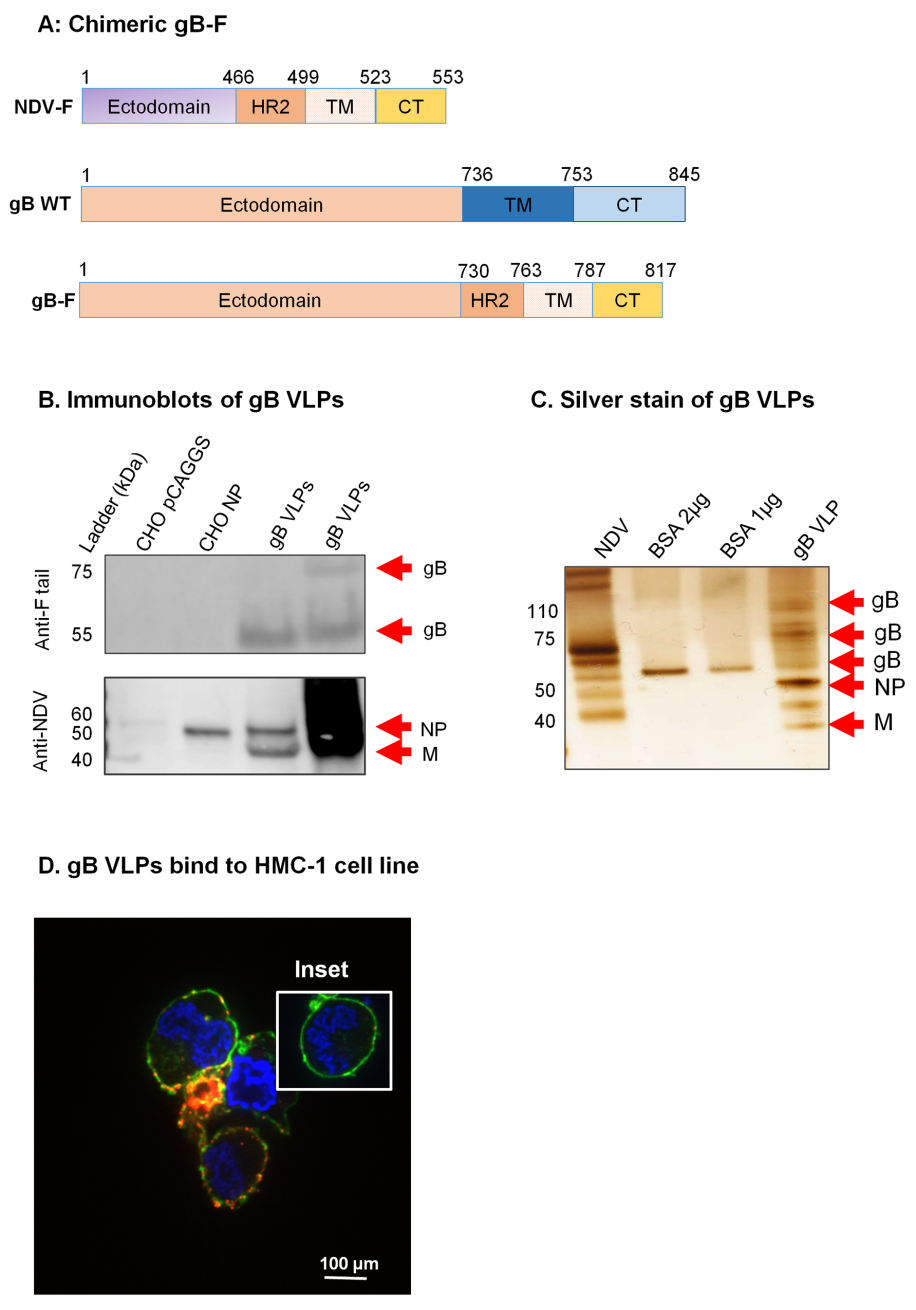
Construction of KSHV gB-F and characterization of KSHV gB VLPs **A**. KSHV gB-F plasmid constructs (not to scale) showing full-length NDV-F (top), full-length/wild-type (WT) gB (middle), and chimeric gB-F (bottom). **B**. CHO cells transfected with empty pCAGGS vector or pCAGGS-NP, or purified gB VLPs were lysed and analyzed by immunoblot using polyclonal anti-NDV F tail and anti-NDV. Anti-F tail (top panel) detected the cleaved forms of the gB-F fusion protein (˜55 and ˜75 kDa) in 5 μg and 10 μg of purified gB VLPs (lanes 3-4), but not in CHO pCAGGS or CHO NP lysates (negative controls, lanes 1-2). Anti-NDV (bottom panel) detected NP alone in CHO NP (positive control, lane 2), and NP and M in gB VLPs (lanes 3-4), but not in CHO pCAGGS (negative control, lane 1). **C**. Silver stain was used to visualize VLP purity relative to purified NDV, and detected uncleaved and cleaved gB-F proteins of ˜110, and 55-75 kDa. Arrows indicate viral/VLP components in 5 μl purified NDV diluted 1:30 (lane 1), and 1 μl purified gB VLPs diluted 1:40 (lane 4). Lanes 2 and 3 were loaded with 1 μg and 2 μg BSA, respectively for protein quantification. **D**. Confocal microscopy shows gB VLPs binding to surface receptors on HMC-1 cells. Purified gB VLPs were incubated with HMC-1 cells for 10 min at room temperature and stained with rabbit polyclonal anti-gB, followed by secondary antibody conjugated with Alexa-Fluor 594 (red) to detect gB VLPs, cholera toxin (green) to detect lipid rafts, and DAPI (blue) to detect HMC-1 nuclei. The red and green staining shows VLPs bound cell membranes; HMC-1 cells not incubated with VLPs did not stain red (negative control, inset).

Upon successful generation and characterization of gpK8.1 VLPs, we used a similar strategy to generate KSHV gB and KSHV gH/gL VLPs. To generate KSHV gB VLPs, we synthesized a chimeric gB-F construct by fusing the KSHV gB ectodomain to the NDV-F HR2, TM, and CT domains (Figure 3A). The construct was cloned into the pCAGGS expression vector, sequence verified, and used to co-transfect CHO cells, together with equal amounts of NDV-NP and NDV-M. KSHV gB VLPs were purified as previously described [25, 36]. We confirmed expression of component proteins gB, NDV-M, and NDV-NP using immunoblot of purified gB VLPs and relevant controls. Due to lack of an effective commercial antibody that has been optimized against KSHV gB, we detected the gB-F fusion protein using anti-NDV F tail. Anti-F tail predominantly detected the cleaved forms of the gB-F fusion protein (˜75 and ˜55 kDa). Anti-NDV detected NDV-NP in CHO cells transfected with pCAGGS-NP (positive control), and NP and M in gB-F VLPs, but not in CHO cells transfected with empty pCAGGS vector (negative control) (Figure 3B).

To assess purity, and to confirm detection of the various components of the gB VLP, we used silver stain. NDV lysate was used as a positive control, and BSA was used for quantification of protein. KSHV gB is synthesized as a 110-kDa precursor protein which undergoes furin cleavage and processing to yield envelope-associated disulfide-linked 75- and 55-kDa polypeptides [37]. Bands of predicted sizes were detected for uncleaved (˜110 kDa) and furin-cleaved gB (˜75 and 55 kDa), NDV-NP (˜50 kDa), and NDV-M (35-45 kDa) (Figure 3C). To confirm that gB-F proteins incorporated on the surface of VLPs were functional and bound cell surface receptors, we incubated purified gB VLPs with HMC-1 cells and performed immunofluorescent confocal microscopy, as above. Cells incubated in the presence, but not the absence (inset), of gB VLPs showed binding of VLPs (red) with lipid rafts in cell membranes (green), indicative of functional binding (Figure 3D).

**Figure 4:**
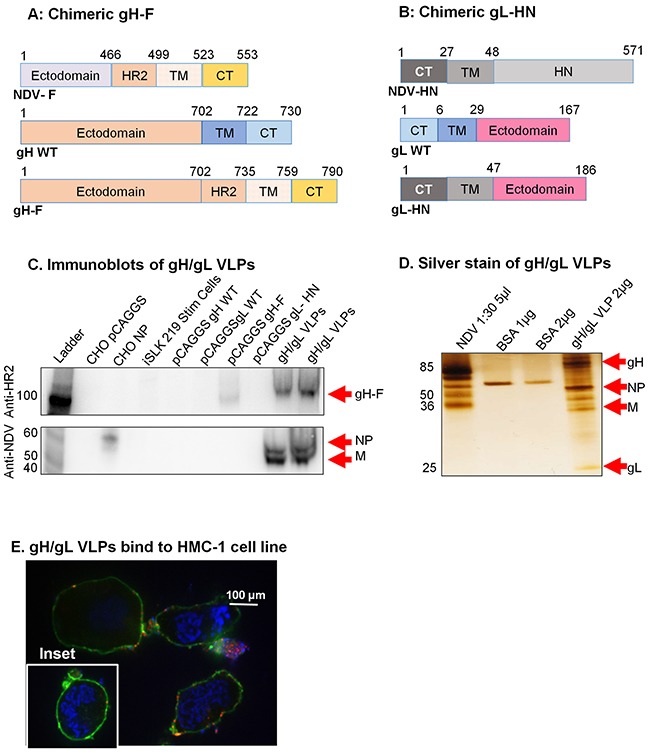
Construction of KSHV gH-F/gL-HN and characterization of KSHV gH/gL VLPs **A**. gH-F plasmid constructs (not to scale) showing full-length NDV-F (top), full-length/wild-type (WT) gH (middle), and chimeric gH-F (bottom). **B**. gL-HN plasmid constructs (not to scale) showing full-length NDV-HN (top), full-length/WT gL (middle), and chimeric gL-HN (bottom). **C**. Purified gH/gL VLPs, CHO cells transfected with pCAGGS, pCAGGS-NP, -gH WT, -gL WT, -gH-F, gL-HN, and lytically induced iSLK KSHV-eGFP expressing cells were lysed in RIPA buffer and analyzed by immunoblot with polyclonal anti-NDV F HR2 and anti-NDV. Anti-HR2 detected gH-F in 5 μg and 10 μg gH/gL VLPs (lanes 9-10) and in CHO gH-F cells (positive control, lane 7), but not other samples (negative controls). Anti-NDV (bottom panel) detected NP alone in CHO NP (positive control, lane 3), and NP and M in gB VLPs (lanes 9-10), but not in other samples (negative controls). For detection of gL-HN using polyclonal antibody raised from mice immunized with gH/gL VLPs see Supplementary Figure 2. **D**. Silver stain was used to visualize VLP purity relative to purified NDV. Arrows indicate viral/VLP components in 5 μl purified NDV diluted 1:30 (lane 1), and 2 μl purified gH/gL VLPs diluted 1:40 (lane 4). Lanes 2 and 3 were loaded with 1 μg and 2 μg BSA, respectively for protein quantification. **E**. Confocal microscopy shows gH/gL VLPs binding to surface receptors on HMC-1 cells. Purified gH/gL VLPs were incubated with HMC-1 cells for 10 min at room temperature and stained with anti-gH, followed by secondary antibody conjugated with Alexa-Fluor 594 (red) to detect gH/gL VLPs, cholera toxin (green) to detect lipid rafts, and DAPI (blue) to detect HMC-1 nuclei. The red and green staining shows VLPs bound cell membranes; HMC-1 cells not incubated with VLPs did not stain red (negative control, inset).

To generate KSHV gH/gL VLPs, we synthesized chimeric gH-F and gL-HN constructs by fusing the KSHV gH ectodomain to the NDV-F HR2, TM, and CT domains (Figure 4A), and the KSHV gL ectodomain to the NDV hemagglutinin-neuraminidase protein (NDV-HN) TM and CT domains (Figure 4B). The rationale for fusion of a type 1 membrane protein (i.e., gH) to NDV-F protein and a type 2 membrane protein (i.e., gL) to NDV-HN has been described [38]. Synthesized cDNA constructs (gH-F and gL-HN) were individually cloned into the pCAGGS expression vector, sequence verified, and used to co-transfect CHO cells with NDV-NP and -M. VLPs were purified as previously described [25, 36]. To confirm protein components of purified VLPs we performed immunoblot analysis with purified VLPs lysed in RIPA buffer (Figure 4C). Due to lack of an effective commercial antibody optimized against KSHV gH/gL for immunoblot, we detected the gH-F chimeric protein using anti-NDV F HR2 domain (top panel). Anti-HR2 detected the gH-F component in purified gH/gL VLPs (5 μg and 10 μg), but not in CHO cells transfected with empty pCAGGS vector, pCAGGS NDV-NP, or wild-type gH or gL, or in lysates from lytically induced iSLK.219 KSHV-eGFP-expressing cells (negative controls). Anti-NDV detected NDV-NP and -M on gH/gL VLPs and NP alone in CHO cells transfected with pCAGGS-NP (positive control), but not in CHO cells transfected with empty pCAGGS vector, CHO cells transfected with wild-type gH, gL, or chimeric gH-F, or in iSLK.219 lysate (negative controls) (bottom panel). Further immunoblot analysis of gH/gL proteins using unpurified sera from mice immunized with gH/gL VLPs confirmed VLP composition to include gH, gL, NP and M (Supplementary Figure 2).

To assess purity of the gH/gL VLPs, and to confirm expression of the gH and gL protein components, we used silver stain. NDV lysate was used as a positive control, and BSA (1 μg and 2 μg) was used for quantification of protein. Bands of predicted sizes were detected for gH (85 kDa), gL (25 kDa), NDV-NP (˜50 kDa), and NDV-M (35-45 kDa) (Figure 4D). To confirm that the VLPs were functional and bound cell surface receptors, we incubated purified gH/gL VLPs with HMC-1 cells and performed immunofluorescent confocal microscopy, as above. Cells incubated in the presence, but not the absence (inset), of gH/gL VLPs showed binding of VLPs (red) with lipid rafts in cell membranes (green), indicative of functional binding (Figure 4E).

### KSHV VLPs stimulate KSHV-specific IgG antibody responses in immunized BALB/c mice

To test the ability of individual KSHV envelope glycoprotein-based VLPs to stimulate KSHV-specific antibody responses, three groups of five female BALB/c mice each were immunized intraperitoneally with 10 μg of purified gpK8.1, gB, or gH/gL VLPs suspended in 0.5 ml of TNE buffer without adjuvants. Two groups of five mice each were immunized with TNE buffer alone (negative control) or UV-inactivated KSHV (positive control). To test additive or synergistic effects of two or more KSHV glycoprotein immunogens [39], we also immunized four groups of five mice each with two or three individual VLPs (gpK8.1 and gB; gpK8.1 and gH/gL; gB and gH/gL; or gpK8.1, gB, and gH/gL).

**Figure 5:**
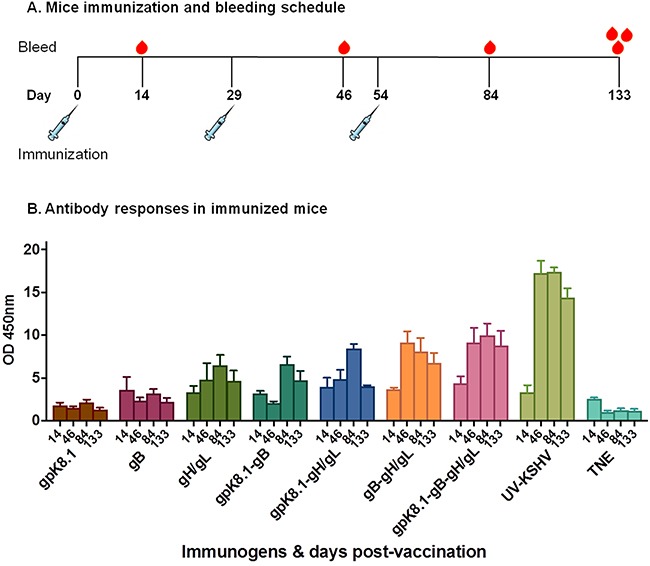
Immunization and generation of specific anti-KSHV IgG antibody responses in BALB/c mice **A**. Immunization and bleeding schedule of 6–8-week-old female BALB/c mice. A total of 9 groups of five BALB/c mice each were immunized with 10 μg of purified gpK8.1, gB, or gH/gL VLPs, or a combination of gpK8.1 and gB; gpK8.1 and gH/gL; gB and gH/gL; or gpK8.1, gB, and gH/gL VLPs. Two groups were immunized with either 10 μg of purified UV-inactivated KSHV (positive control) in TNE buffer, or TNE buffer alone (negative control). The immunizations were administered on Day 0 (primary), followed by two boosts on Days 29 and 54, respectively. Mice were tail-vein bled on Days 14, 46, and 84, followed by terminal bleed on Day 133. **B**. Antibody titers in sera from immunized BALB/c mice were determined by ELISA using lysate from lytically induced iSLK.219 KSHV-eGFP-expressing cells as target antigen. Serum samples from Days 14, 46, 84, and 133 diluted 1:100 in PBS were incubated in microtiter plates coated with iSLK.219 KSHV-eGFP cell lysate. Lysates from iSLK cells (free of KSHV infection) and irrelevant ELL-0 cell lines were used as negative controls. Antibody binding was detected using HRP-labeled goat anti-mouse IgG secondary antibody. The absorbance resulting from serum antibody binding to KSHV-eGFP-expressing iSLK.219 cell lysate target antigen is shown as summary data for each group of mice. Absorbance data are shown as the mean (top of bar) ± the SEM (error bar above bar) for five mice per group (see Supplementary Figure 3 for individual mice at 2 time-point). KSHV VLPs induced increasing titers of anti-KSHV specific IgG antibodies after each immunization.

The mice were immunized at Day 0 and boosted twice, on Days 29 and 54. Mice were tail-vein bled on Days 14, 46, and 84, with a terminal bleed on Day 133 (Figure 5A).

To demonstrate the ability of KSHV VLPs to induce KSHV-specific antibody responses, we used ELISA to determine the presence of anti-KSHV IgG antibodies in sera of immunized mice. Lysate from lytically induced iSLK.219 KSHV-eGFP-expressing cells, which express all KSHV envelope glycoproteins, served as the binding target. Sera from all bleeding time-points were analyzed. All VLP-immunized mice generated KSHV-specific IgG antibody responses that increased following booster immunizations, compared with TNE-immunized mice (negative control) (Figure 5B). The increase in KSHV-specific antibody response peaked by Day 84, after the second booster immunization, similarly to UV-inactivated KSHV-immunized mice (positive control). The antibody responses to the KSHV-envelope glycoprotein-based VLPs were long-lived, as the titers were still high even after 133 days. A detailed analysis of serum antibody responses in individual mice at each time point is provided in Supplementary Figure 3.

### Antibodies from mice immunized with KSHV VLPs neutralize KSHV infection *in vitro*

**Figure 6:**
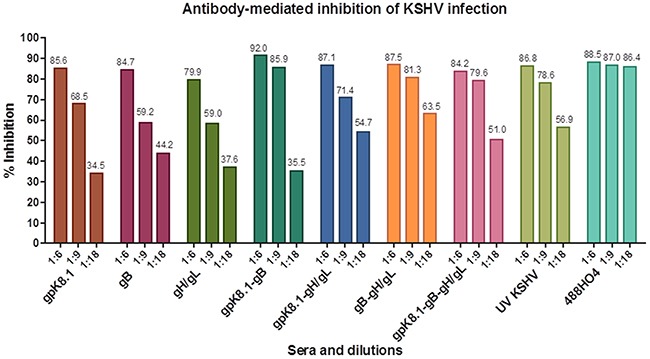
Neutralization assay of KSHV-eGFP with sera from mice immunized with KSHV VLPs Pooled heat-inactivated terminal bleed sera from 5 animals per immunization treatment was pre-incubated with KSHV-eGFP purified from lytically induced iSLK.219 cells, then incubated at 37°C for 2 h with HEK-293 cells. The virus/serum inoculum was removed and replaced with 10% DMEM, and the HEK-293 cells further incubated at 37°C for 48 h. KSHV-eGFP+ cells, indicative of infection, were enumerated by flow cytometry. Known KSHV-positive plasma (488HO4; a gift of Dr. Wood University of Nebraska, Lincoln) and sera from UV-KSHV immunized mice served as positive controls Human sera 488HO4 neutralized ˜80% of KSHV infection at 1:6, 1:9, and 1:18 dilutions. Sera from mice immunized with TNE (not shown) served as negative control and was used to normalize the percent infection. All the groups of mice immunized with single VLPs or different combinations of VLPs generated a neutralizing antibody response against KSHV-eGFP. At a 1:18 serum dilution, pooled serum from individual mice immunized with a combination of either gpK8.1-gH/gL, gB-gH/gL, or gpK8.1-gB-gH/gL VLPs showed the best neutralization, inhibiting infection by 54.7%, 63.5%, and 51.0% respectively, compared to <50% for the mice immunized with single gpK8.1, gB, or gH/gL VLPs.

To assess the ability of VLP-immunized sera to neutralize KSHV infection *in vitro*, sera collected at Day 133 were used to conduct KSHV infection neutralization assays. HEK-293 cells were used as the host for the neutralization assays, as a model of epithelial cells that are susceptible to KSHV infection [40]. Cells were exposed to KSHV-eGFP purified from lytically induced iSLK.219 cells in the absence or presence of sera from VLP-immunized animals. The virus titer was determined by an infectivity assay, as previously described [25] and as briefly outlined in Materials and Methods sections. Known KSHV-positive plasma (488HO4), a gift of Dr. Wood, University of Nebraska, Lincoln, served as positive control and neutralized >80% of KSHV infection at 1:6, 1:9, and 1:18 dilutions. Sera from mice immunized with TNE (data not shown) served as negative control and was used to normalize the percent infection. Upon pre-incubation of 60 μl of KSHV-eGFP with sera from mice immunized with KSHV glycoprotein-based VLPs, infection of HEK-293 cells was reduced with increasing antibody concentration (Figure 6). At a 1:18 serum dilution, pooled serum from individual mice immunized with a combination of either gB-gH/gL, gpK8.1-gB-gH/gL, or gpK8.1-gH/gL VLPs showed the best neutralization, inhibiting infection by 63.5%, 51%, and 54.7% respectively, compared to <50% for the mice immunized with single gpK8.1, gB, or gH/gL VLPs.

**Figure 7:**
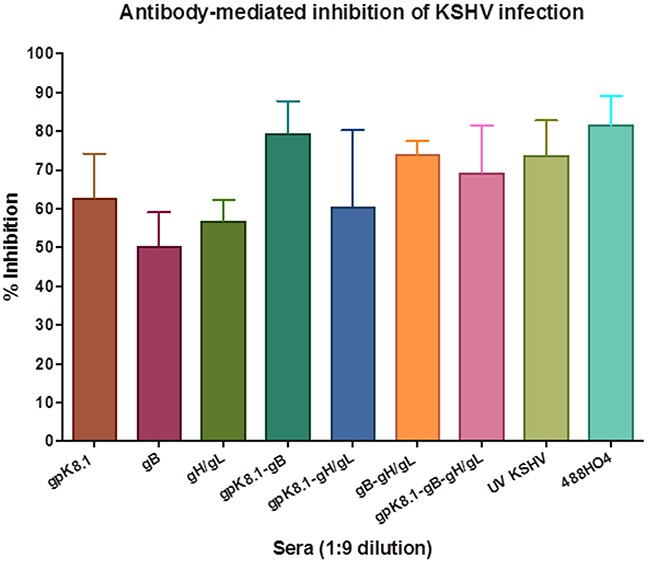
Neutralization assay of KSHV-eGFP+ with 1:9 diluted sera from mice immunized with KSHV VLPs Neutralization assays were performed as described in Figure 6 and Materials and Methods. Data from three independent experiments at a 1:9 serum dilution is presented. The percent inhibition of infection is shown as the mean (top of bar) ± the SEM (error bar above bar) for each serum group. Known KSHV positive plasma (488HO4) served as positive control and neutralized >80% of KSHV infection. Serum from mice immunized with TNE (data not shown) served as negative control and was used to normalize the percent infection. As a single immunogen, gpK8.1 VLPs stimulated comparable neutralizing antibody activity to that of UV-inactivated KSHV (positive control). In contrast, UV-inactivated KSHV stimulated higher titers of neutralizing antibodies compared to gB (p = 0.0316) or gH/gL (p = 0.0486). Mice immunized with the combination of gB and gH/gL VLPs had a better neutralizing antibody response than those immunized with either gB (p = 0.0268), or gH/gL (p = 0.0397) VLPs as single immunogens. Immunization with all VLP combinations containing gpK8.1 (gpK8.1-gB, gpK8.1-gH/gL and gpK8.1-gB-gH/gL) stimulated comparable neutralizing antibody activity to UV-KSHV serum.

Data from three independent experiments at a 1:9 serum dilution were used to determine the effectiveness of each glycoprotein in eliciting neutralizing antibodies as a single or combined immunogen(s) (Figure 7). Sera from mice immunized with a gpK8.1 VLPs as a single immunogen stimulated comparable neutralizing antibody activity to UV-inactivated KSHV. In contrast, immunization with gB or gH/gL VLPs as single immunogens stimulated a less effective neutralizing antibody responses compared to UV inactivated-KSHV (p = 0.0316, p = 0.0486, respectively). Immunization with a combination of gB and gH/gL VLPs had a better neutralizing antibody response than either single immunogen, gB (p = 0.0119), or gH/gL (p = 0.0133). Importantly, immunization with any combination containing gpK8.1 VLPs (gpK8.1-gB, gpK8.1-gH/gL, and gpK8.1-gB-gH/gL) stimulated comparable neutralizing antibody activity to UV-inactivated KSHV. Our findings confirm that immunization with a combination of immunogens has an additive effect on immunogenicity and neutralizing antibody response.

## DISCUSSION

KS is a significant cause of morbidity and mortality in sub-Saharan Africa. Primary prevention by vaccination against KSHV could play an important role in limiting virus shedding and transmission, thus reducing the burden of KS in KSHV-endemic areas. To the best of our knowledge, this is the first study to generate VLPs using KSHV glycoproteins gpK8.1, gB, or gH/gL as target immunogens to serve as prophylactic vaccine candidates for prevention of KSHV infection.

We showed that co-transfection of CHO cells with chimeric KSHV envelope glycoproteins and NDV structural proteins leads to successful production of gpK8.1, gB, and gH/gL VLPs. These VLPs resemble native virus and bind the KSHV-susceptible HMC-1 cell line, demonstrating that the conformation of the viral glycoproteins is conserved and functional.

Importantly, immunization of female wild-type BALB/c mice with KSHV glycoprotein-based VLPs singly or in combination induced humoral immune responses, resulting in moderately high titers of serum antibodies relative to UV-inactivated KSHV. Antibody titers peaked after the second boost immunization and did not significantly decrease until Day 133, suggesting that VLPs can induce long-term immunogenicity in mice. This also provides circumstantial evidence that memory plasma cells are developed in response to vaccination with KSHV glycoprotein-based VLPs.

UV-inactivated KSHV induced higher titers of KSHV-specific IgG antibodies than KSHV glycoprotein-based VLPs administered singly or in combination, suggesting it was a better immunogen. However, the high antibody titers detected in response to UV-inactivated KSHV could be due to non-target-specific binding, possibly to UV-inactivated KSHV itself, or due to the presence of other antibodies directed to the iSLK.219 cells used for virus production and as the target lysate in ELISA. Furthermore, whether some threshold level of vaccine-induced antibody titer is required to prevent infection *in vivo* remains to be elucidated in an appropriate animal model. A humanized mouse model [41] and non-human primate model [42] have been developed and characterized to support studies of KSHV infection, and would be ideal for demonstrating *in vivo* efficacy of candidate vaccines in future studies. If increased antibody titers are required to prevent infection *in vivo*, we could increase immunogenicity by administering the KSHV glycoprotein-based VLPs in the presence of adjuvant [43], or by titrating the VLP concentration to achieve an optimal dose to elicit effective KSHV-specific neutralizing antibody titers [44].

Neutralizing antibodies can inhibit progression of herpesvirus-associated diseases [45–47]. Thus, a vaccine that elicits a neutralizing antibody response could help prevent KSHV infection and its associated malignancies in KSHV-endemic populations [48]. The KSHV glycoprotein-based VLPs induced neutralizing antibody responses in all immunized mice. The neutralizing antibodies blocked KSHV infection *in vitro* in a dose-dependent manner. Immunization with a single immunogen, gpK8.1, induced neutralizing antibody activity that was comparable to UV-inactivated KSHV, the gold standard. Immunization with a combination of gB and gH/gL VLPs induced a better neutralizing antibody response than either immunogen on its own. Importantly, combination of gpK8.1 with any other KSHV glycoprotein-based VLPs (gpK8.1-gB, gpK8.1-gH/gL, or gpK8.1-gB-gH/gL) induced a neutralizing antibody response that was comparable to that of UV-KSHV. This demonstrates the additive effect of combining more than one immunogen in a potential vaccine, and confirms that gpK8.1 is an important immunogen to include in the vaccine. We are currently developing a polyvalent VLP that expresses all four glycoproteins (gpK8.1, gB, and the gH/gL complex) on the surface of a single VLP. Multivalent VLPs are known to induce higher immunological responses than corresponding monovalent VLPs [49, 50]. A single, multivalent VLP would also be more cost-effective to produce in large-scale.

All herpesviruses persist for life in infected individuals, which means that only complete eradication of the latent virus can cure infection. Thus, our ultimate goal is to develop a vaccine that elicits both humoral and cellular responses to limit viral infection and eradicate infected cells. To elicit a cellular immune response in addition to the humoral response, future KSHV glycoprotein-based VLPs should also incorporate intracellular KSHV T-cell antigens, such as latent nuclear antigen-1 (LANA1; ORF73). LANA1 is responsible for maintaining KSHV as an episome in infected cells, while the virus undergoes latent replication [51]. LANA1 is expressed in all KSHV-infected cells, including KS tumor cells, and is a target of the cellular immune response mediated by CD4+ and CD8+ T cells [52]. LANA1-specific T cells are effective in controlling growth of KSHV-immortalized endothelial and B cells [53, 54]. Therefore, we expect that a VLP comprised of gpK8.1-gB-gH/gL and LANA1 would elicit both humoral and cell-mediated immune responses in immunized hosts. This dual response would allow the VLP vaccine to provide both a prophylactic and therapeutic effect; thus, it could be used to both prevent and treat KSHV and KS in endemic areas. The inclusion of other latent KSHV proteins, such as v-Cyclin (ORF72), v-FLIP (K13 or ORF71), Kaposin (K12), and viral miRNAs, which are also constitutively expressed from the latency locus of the viral genome [55], should be considered as part of a polyvalent KSHV vaccine. A polyvalent vaccine incorporating multiple KSHV glycoproteins and latent proteins could allow tailored targeting of KSHV-associated tumors as a therapeutic treatment strategy.

### Study limitations

*In vivo*, KSHV typically infects epithelial, endothelial, and B cells [56]. Although we showed neutralization of infection in an epithelial cell model, due to limited amounts of mouse sera, we were unable to test the ability of the VLP-generated neutralizing antibodies to block virus infection in endothelial and B cells. Future studies could test this in primary cells or in cell lines susceptible to KSHV infection, such as MC116 [57].

## CONCLUSIONS

We have generated the first KSHV subunit vaccines potentially capable of preventing KSHV infection *in vivo* and thus possibly capable of preventing KS. KS continues to be a major public health concern in sub-Saharan Africa, where KSHV is endemic, and large numbers of HIV-infected individuals have late or no access to HAART [58]. A recent review on the burden of cancer associated with infectious agents listed KS as the second largest cancer burden in sub-Saharan Africa, behind only cervical cancer [59]. The accuracy of clinical and histopathologic diagnosis of KS in low-resource settings such as sub-Saharan Africa is sub-optimal [60]; thus, prevention is the best way to limit KS morbidity and mortality. A prophylactic vaccine to prevent KSHV and KS is critical to reduce the burden of disease in these settings. In endemic areas, KSHV infection is acquired mainly through horizontal transmission, most likely during childhood [4, 48]. A comprehensive prospective study evaluating and comparing primary infection with multiple herpesviruses in a cohort of African infants beginning at birth showed that despite endemicity of KSHV in the region, no KSHV infection was observed in the first 18 months after birth [61]. Instead, other herpesviruses were acquired first. This evidence of delayed KSHV infection suggests that an 18-month window of opportunity exists, in which to vaccinate children and curb KSHV incidence and transmission. Furthermore, seroprevalence of KSHV is low in many developed countries; thus, an effective prophylactic vaccine will be an important tool in eradicating the rare cases of KS that occur during immunosuppression, especially in organ transplant or AIDS patients.

## MATERIALS AND METHODS

### Ethics statement

Animal procedures were performed in accordance with the University of Massachusetts Medical School (UMMS) Institutional Animal Care and Use Committee and Institutional Biosafety Committee.

### Cell lines and virus

Chinese hamster ovary (CHO), human embryonic kidney (HEK-293), East Lansing Line 0 (ELL-0), and human mast (HMC-1) cell lines were purchased from the American Type Culture Collection (ATCC). Parent iSLK and iSLK.219 cells harboring KSHV expressing a reporter gene, enhanced green fluorescence protein, (eGFP), from the human elongation factor (EF)-1α promoter [62] were obtained from Dr. D. Dittmer of University of North Carolina, Chapel Hill, NC. CHO, HEK-293, and ELL-0 cell lines were cultured in Dulbecco's Modified Eagle's Medium (DMEM). HMC-1 cell line was cultured in Iscove's Modified Dulbecco's Medium. All cell culture media contained 10% heat-inactivated fetal bovine serum (FBS), 2% penicillin-streptomycin, and 1% L-glutamine. Media for culturing iSLK.219 cells was supplemented with neomycin (250 μg/mL), hygromycin (400 μg/mL), and puromycin (10 μg/mL) to maintain stable selection of both recombinant KSHV-eGFP and the RTA gene under the pRetro-X Tet-ON inducible system.

Lytic induction of KSHV-eGFP expression in iSLK.219 cells and KSHV-eGFP virus purification were performed as previously described [62]. Purified NDV B1 strain (lysate) was a gift of Dr. T. Morrison (UMMS, Worcester).

### Antibodies and cholera toxin B

Primary mouse monoclonal IgG_2a_ anti-gpK8.1 (clone 4A4), which detects the ectodomain of the protein was purchased from Santa Cruz. Rabbit polyclonal anti-NDV and anti-NDV F HR2 have been described and were gifts of T. Morrison of UMMS, Worcester, MA [63]. Rabbit polyclonal anti-NDV F-tail was raised against a synthetic peptide (YKQKAQQKTLLWLGNN) with the sequence of the cytoplasmic domain of the fusion protein, (amino acids 527 to 543) and purified by Thermo Scientific. Rabbit polyclonal anti-gB and anti-gH used in HMC-1 cell binding experiments were gifts of Dr. B. Chandran of Rosalind Franklin University, Chicago, IL. KSHV-positive human plasma (488HO4) was obtained from Dr. C. Wood, University of Nebraska-Lincoln, NE [48]. Secondary antibodies horseradish peroxidase (HRP)-conjugated goat anti-mouse IgG and goat anti-rabbit IgG antibodies for immunoblot and ELISA were purchased from Sigma. Goat Fab-2 anti-mouse IgG (H+L) conjugated to AF488 or AF594, used for cytometric and fluorescence microscopy analyses, and molecular probe cholera toxin B conjugated with AF488, for staining lipid rafts, were all purchased from Thermo Scientific.

### Plasmid vectors

Individual chimeric fragments consisting of amino acids (aa) 1-197, 1-736, and 1-702 encoding the gpK8.1, gB, and gH ectodomains, respectively, were constructed and fused to the NDV-F heptad repeat (HR2), transmembrane (TM), and cytoplasmic (CT) domains (aa 466-553). A fragment encoding the TM and CT domains of the NDV-HN protein (aa 1-48) was fused to the ectodomain of gL (aa 29-167). The chimeric cDNAs were synthesized by Genewiz, Boston, MA, and individually cloned into the pCAGGS mammalian expression vector [64] to generate pCAGGS-KSHV gpK8.1-F, pCAGGS-KSHV gB-F, pCAGGS-KSHV gH-F, and pCAGGS-KSHV gL-HN. Full-length gpK8.1 wild-type (WT) (aa 1-228), gB WT (aa 1-845), gH WT (aa 1-730), and gL WT (aa 1-167) were also synthesized and individually cloned into pCAGGS vector for use in control experiments. pCAGGS-F, pCAGGS-M, and pCAGGS-NP derived from NDV have been described [38]. All the cloned cDNAs were sequenced to confirm their fidelity.

### gpK8.1 transfection and flow cytometry analyses

To determine surface expression of both wild-type and chimeric KSHV gpK8.1 envelope glycoprotein, 80% sub-confluent CHO cells seeded in six-well tissue culture plates were transfected with 1 μg/well of pCAGGS, pCAGGS-KSHV gpK8.1 full-length (WT), or pCAGGS-KSHV gpK8.1-F using Mirus reagent (Mirus Bio LLC), according to the manufacturer's instructions. Cells were harvested 48 h post-transfection, washed twice by centrifugation at 335 rcf for 5 min, and re-suspended in PBS. The cells were incubated with anti-gpK8.1 mAb for 10 min at room temperature, washed twice, re-incubated with goat anti-mouse AF488, and washed thrice.

Cytometric analysis for gpK8.1 protein surface expression was performed on an LSRII benchtop FC (Becton-Dickinson, BD) and data was analyzed using Flow Jo Cytometry Analysis software (Tree Star Inc) as previously described [25]. A minimum of 10,000 events was recorded for each analysis. Experiments were repeated at least thrice and representative data is presented. Cell surface expression of gB-F, gB-WT, gH-F, gH-WT, gL-HN, gL-WT, or gH-F/gL-HN were not determined by flow cytometry due to lack of primary monoclonal antibodies for staining transfected cells.

### Generation and purification of VLPs

For VLP preparation, equal amounts (8 μg/plasmid) of pCAGGS-NDV-M, -NP, and either pCAGGS-KSHV gpK8.1-F, -gB-F, or -gH-F and -gL-HN (for KSHV gpK8.1, gB, and gH/gL VLPs, respectively) were co-transfected into CHO cells seeded in T-175 cm^2^ flasks, using Mirus reagent, as previously described [25]. Forty flasks were seeded for each VLP preparation. Supernatant containing VLPs was collected between 24 to 96 h post-transfection. VLPs were isolated by sedimentation and sucrose gradient purification, as previously described [36].

### Silver stain and immunoblot

To detect the incorporation of proteins into VLPs, purified KSHV glycoprotein-based VLPs, untransfected CHO cells (negative control), and cells transfected with the respective wild-type proteins (positive controls) were first lysed in RIPA buffer (Boston Bioproducts). Lysates were incubated on ice for 30 min, with vortexing every 10 min, then centrifuged for 10 min at 18,407 rcf in a microcentrifuge. The protein content of each lysate was determined by Bradford assay (Sigma). Lysates were boiled for 5 min in lithium dodecyl sulphate sample buffer (Thermo Scientific) under reducing and non-reducing conditions. A known quantity of protein lysate was loaded onto a 4–12% polyacrylamide gel for protein separation using 1X MES SDS running buffer (Life Sciences Technologies). Immunoblot analyses for respective proteins (gpK8.1, gB, gH/gL, NDV-NP, and -M) were performed by transferring proteins from the gel to a polyvinylidene fluoride membrane using iBlot (Life Sciences Tech). Membranes were blocked with 3% bovine serum albumin (Sigma) in Tris-buffered saline (TBS) for 45 min and proteins were detected with specific primary and secondary antibodies as previously described [65, 66]. Protein bands were also detected by Pierce's silver stain kit according to the manufacturer's recommendation.

### Cell binding assays and electron microscopy

VLPs and viruses were analyzed by electron microscopy as described [67]. For cell binding assays, 5 μl of KSHV glycoprotein-based purified VLPs (gpK8.1, gB, or gH/gL) were incubated with HMC-1 cells for 10 min at room temperature and detected with anti-gpK8.1 mAb, polyclonal anti-gB, or polyclonal anti-gH. Lipid rafts in the cell membranes were stained with cholera toxin (cell membrane tracker) and nuclei were stained with 4’,6-diamidino-2-phenylindole (DAPI). For electron microscopy, purified VLPs were dialyzed against 1 L of TNE buffer (100 mM Tris; 2.0 M NaCl; 10 mM EDTA; pH 7.4) to remove residual sucrose, incubated with 3% bovine serum albumin (BSA) in TNE for 45 min, and embedded on a grid. 5 μl of the virus/VLP at 1:40 dilution was individually added to the grid for 1 h at room temperature. After two final washes, the grids were negatively stained with 12% phosphotungstic acid (pH 7) for 15 sec, air dried for 30 min, and examined using a Tecnai transmission electron microscope (FEI).

### Immunization

Nine groups of five 6–8-week-old female BALB/c mice (Jackson Laboratories) each were immunized intraperitoneally with 10 μg of either purified KSHV gpK8.1 VLP, gB VLP, gH/gL VLP, or combinations of gpK8.1 and gB, gpK8.1 and gH/gL, gB and gH/gL, or gpK8.1 and gB and gH/gL VLPs in 0.5 mL TNE buffer containing ˜10% sucrose without any adjuvant. UV-inactivated KSHV-eGFP purified from lytically induced iSLK.219 cells (10 μg in 0.5 mL TNE) or TNE buffer alone (0.5 mL) served as positive and negative controls, respectively. UV-inactivation of KSHV was achieved by exposure to 254 nm UV light for 5 min (source model UVG-11, UVP) from a distance of 10 cm, producing complete loss of eGFP expression upon KSHV infection of 293T cells.

Mice were boosted with 10 μg of each antigen on Days 29 and 54. After primary immunization, tail vein blood was obtained at Days 14, 46, and 84. A terminal bleed was obtained on Days 133. All time points were used to determine anti-KSHV glycoprotein IgG response.

### Enzyme-linked immunosorbent assay (ELISA) to determine sera antibody titer

Anti-KSHV IgG absorbance was measured by ELISA using cell lysate from iSLK.219 cells stimulated to induce lytic expression of KSHV-eGFP proteins as a target antigen. Lysates from iSLK cells (free of KSHV infection) and irrelevant ELL-0 cell lines were used as negative controls. Briefly, 96-well microtiter plates (Costar) were coated overnight with 8 μg/mL per well of lytically induced iSLK.219 KSHV-eGFP-expressing cell lysate in PBS at 4°C, then blocked with 2% BSA at room temperature. Serum samples diluted 1:100, 1:300, 1:900, and 1:2700 in PBS were added, incubated for 2 h at room temperature, then washed thrice. Antibody binding was detected with HRP-labeled goat anti-mouse IgG secondary antibody incubated at room temperature for an hour. Plates were washed five times and the substrate ABTS (KPL) was added. Reactions were stopped with ABTS peroxidase stop solution (KPL). Optical density (OD) was read at 450 nm with an ELISA reader (FilterMax F3) and corrected for background. The absorbance resulting from serum antibody binding to KSHV-eGFP-expressing iSLK.219 cell lysate target antigen is shown as summary data for each group of mice. Absorbance data at 1:100 are provided as the mean (top of bar) ± the SEM (error bar above bar) for five mice per group. KSHV VLPs induced increasing titers of anti-KSHV specific IgG antibodies after each immunization.

### KSHV neutralization studies

Terminal bleed sera from each group of immunized mice were pooled and incubated at 56°C for 30 min to heat-inactivate serum complement. The virus titer for the *in vitro* neutralization assays was determined as described [25]. Briefly, different concentrations of a frozen stock of KSHV-eGFP purified from lytically induced iSLK.219 cells were incubated with HEK-293 cells seeded overnight in 48-well plates, in the absence of serum. Flow cytometry was used to detect fluorescence, indicative of infection. After infection with 30 μl of stock KSHV-eGFP, ˜15% (1,500 cells) of total seeded HEK-293 cells reproducibly fluoresced upon detection by flow cytometry. For effective flow cytometry reading, the number of HEK-293 cells was increased and seeded in 24-well plates and the amount of virus used for infection was doubled to 60 μl to accommodate the increase in cell population.

Serial two-fold dilutions of heat-inactivated sera were prepared in Dulbecco's modified Eagle's medium (DMEM) without fetal bovine serum to a volume of 60 μl. This serum was incubated with 60 μl KSHV-eGFP for 2 h at 37°C. The serum/virus mixture was topped up with serum-free media to a final volume of 200 μl, yielding dilutions ranging from 1:3 to 1:150, then used to infect HEK-293 cells that had been seeded at 60-80% confluence in 24-well plates the night before. The KSHV-eGFP-incubated sera were cultured with the cells for 2 h at 37°C. Known neutralizing KSHV-positive plasma (488HO4) [48] and serum from TNE only-treated mice served as positive and negative controls, respectively. The virus/serum inoculum was removed and replaced with 500 μl of fresh DMEM plus 10% FBS. Plates were incubated for 48 h at 37°C and the number of eGFP-positive cells was determined by flow cytometry. Neutralization assays were performed in triplicate to determine the optimal serum dilutions for the assays. Evaluation of data to determine the magnitude of neutralization was performed as previously described, and quantified as percent inhibition of viral infection [48]. Briefly, sample data was first normalized to input viral infection and calculated as: Normalized Percent Infectivity = (Sample percent infectivity/Virus-only percent infectivity) x 100. The percent inhibition of viral infection was then calculated as: (100 - Normalized Percent Infectivity).

### Statistical analysis

Graph Pad Prism 6 Software was used for statistical analyses of data. The differences between the percent inhibition of infection between the immunized and control groups of mice were analyzed using unpaired two-tailed t-tests for independent groups. Statistical significance of the tests was based on a p-value equal to or lower than 0.05.

## SUPPLEMENTARY MATERIALS FIGURES


