# Correction: BALB/c mice immunized with a combination of virus-like particles incorporating Kaposi sarcoma-associated herpesvirus (KSHV) envelope glycoproteins gpK8.1, gB, and gH/gL induced comparable serum neutralizing antibody activity to UV-inactivated KSHV

**DOI:** 10.18632/oncotarget.28600

**Published:** 2024-07-02

**Authors:** Anne K. Barasa, Peng Ye, Meredith Phelps, Ganapathiram T. Arivudainambi, Timelia Tison, Javier Gordon Ogembo

**Affiliations:** ^1^Department of Experimental Therapeutics, Beckman Research Institute of City of Hope, Duarte, CA, USA; ^2^Department of Medicine, University of Massachusetts Medical School, Worcester, MA, USA; ^3^Department of Human Pathology, University of Nairobi, Nairobi, Kenya

**This article has been corrected:** Figure 2C, panel I mistakenly shows a TEM image of a different virus (Epstein-Barr Virus) instead of the indicated Kaposi sarcoma associated herpesvirus (KSHV). The corrected Figure 2C, with an accurate KSHV TEM image obtained using the original data, is shown below. The authors declare that this correction does not change the results or conclusions of this paper.

Original article: Oncotarget. 2017; 8:34481–34497. 34481-34497. https://doi.org/10.18632/oncotarget.15605

**Figure 2:**
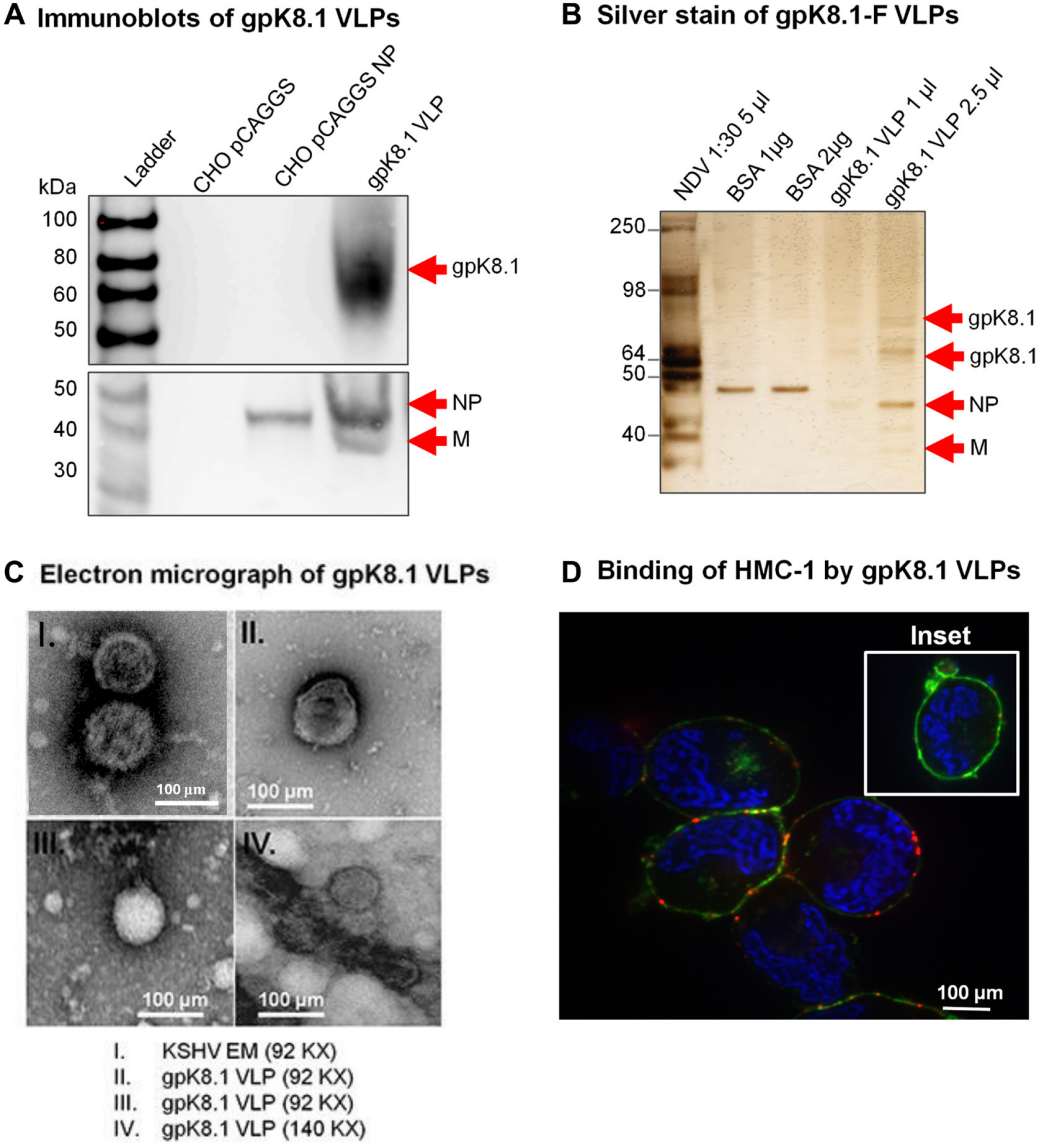
Characterization of KSHV gpK8.1 VLPs. (**A**) CHO cells transfected with empty pCAGGS or pCAGGS-NP, or purified gpK8.1 VLPs were lysed and analyzed by immunoblot using anti-gpK8.1 mAb and rabbit polyclonal anti-NDV to detect various VLP components. Anti-gpK8.1 (top panel) detected gpK8.1 (62-72 KDa) in VLP lysate (lane 4), but not in CHO pCAGGS or CHO NP (negative controls, lanes 2-3). Anti-NDV (bottom panel; a gift of Dr. T. Morrison, University of Massachusetts Medical School) detected NP alone in lysate from CHO NP (lane 3), and NP and M in gpK8.1 VLPs (lane 4), but not in CHO pCAGGS lysate (negative control, lane 2). (**B**) Silver stain was used to visualize VLP purity relative to purified NDV. Arrows indicate viral/VLP components in 5 μl purified NDV diluted 1:30 (lane 1), and 1 μl and 2.5 μl of purified gpK8.1 VLPs diluted 1:40 (lanes 4-5). Lanes 2 and 3 were loaded with 1 μg and 2 μg BSA, respectively for protein quantification. (**C**) Electron microscopy showing structural similarity between KSHV virions and gpK8.1 VLPs. Purified VLPs were dialyzed against 1 L TNE buffer to remove residual sucrose, incubated with 3% bovine serum albumin (BSA) in TNE for 45 min, and embedded on a grid. 5 μl of the virus/VLP at 1:40 dilution was individually added to the grid for 1 h at room temperature. After two final washes, the grids were negatively stained with 12% phosphotungstic acid (pH 7) for 15 sec, air dried for 30 min, and examined using a Tecnai transmission electron microscope (FEI). (**D**) Confocal microscopy shows gpK8.1 VLPs binding to lipid rafts on HMC-1 cells. Purified gpK8.1 VLPs were incubated with HMC-1 cells for 10 min at room temperature and stained with anti-gpK8.1, followed by secondary antibody conjugated with Alexa-Fluor 594 (red) to detect gpK8.1 VLPs, cholera toxin (green) to detect lipid rafts, and DAPI (blue) to detect HMC-1 nuclei. The red and green staining shows VLPs bound cell membranes; HMC-1 cells not incubated with VLPs did not stain red (negative control, inset). VLPs (red) binding to lipid rafts are not seen in HMC-1 cells not incubated with VLPs (negative control, inset).

