# Supplementary material for: BALB/c mice immunized with a combination of virus-like particles incorporating Kaposi sarcoma-associated herpesvirus (KSHV) envelope glycoproteins gpK8.1, gB, and gH/gL induced comparable serum neutralizing antibody activity to UV-inactivated KSHV
Source: Oncotarget. 2017 Feb 22;8(21):34481–97. doi: 10.18632/oncotarget.15605 (PMC5470984; doi:10.18632/oncotarget.15605)
Supplement: Supplementary file 1 [file oncotarget-08-34481-s001.pdf]

BALB/c mice immunized with a combination of virus-like particles incorporating Kaposi sarcoma-associated herpesvirus (KSHV) envelope glycoproteins gpK8.1, gB, and gH/gL induced comparable serum neutralizing antibody activity to UV-inactivated KSHV

SUPPLEMENTARY FIGURES

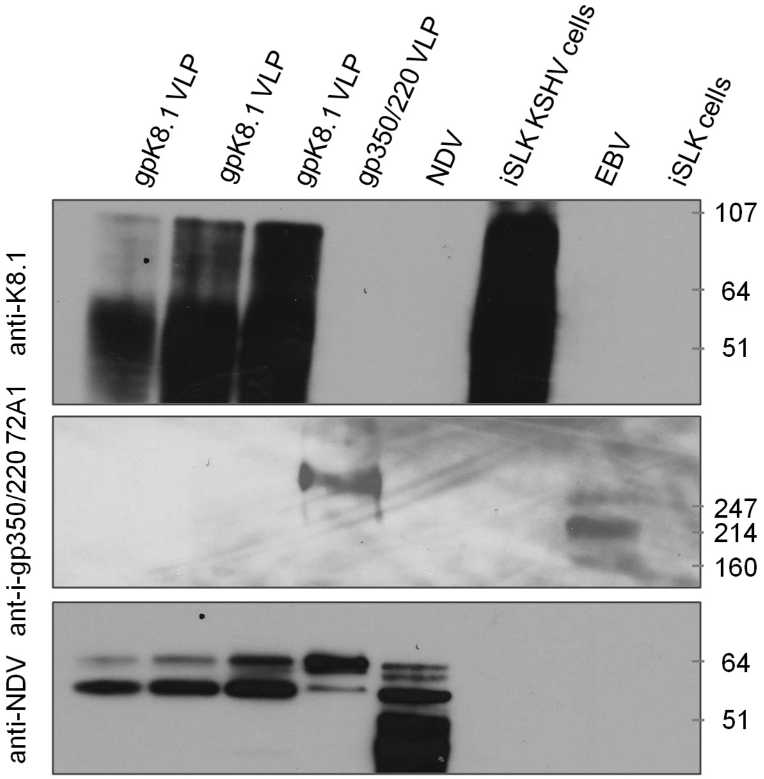

Supplementary Figure 1: Analysis of purified KSHV gpK8.1 VLP by immunoblot. CHO cells were co-transfected with pCAGSS-gpK8.1-F, -NDV NP, and -M plasmids. Supernatants from transfected cells were harvested daily (24-96 h). VLPs in the supernatant were concentrated by centrifugation and purified using sucrose density gradients as described in Materials and Methods. Different concentrations of purified gpK8.1 VLPs, Epstein-Barr VLPs (gp350/220 VLPs), purified NDV, lytically induced iSLK.219 KSHV-eGFP-expressing cells, purified Epstein-Barr virus (EBV, negative control), and iSLK cells were lysed using RIPA buffer and boiled in non-reducing SDS-Laemmli buffer followed by immunoblot. gpK8.1 protein was detected by anti-gpK8.1 mAb (top panel) different concentrations of gpK8.1 VLPs (lanes 1-3) and KSHV-eGFP-expressing iSLK.219 cells (positive control, lane 6). gpK8.1 was not detected in gp350/220 VLPs, purified NDV, purified EBV, or iSLK cells (all negative controls; lanes 4, 5, 7, and 8). Anti-gp350/220 mAb 72A1 (middle panel) detected gp350/220 protein (irrelevant protein not expected in KSHV VLPs) in gp350/220 VLPs (lane 5) and purified EBV (lane 7) only. Polyclonal rabbit anti-NDV (bottom panel) detected NDV-NP and -M components in all lanes loaded with NDV-based VLPs (lanes 1-4) and purified NDV (positive control, lane 5).

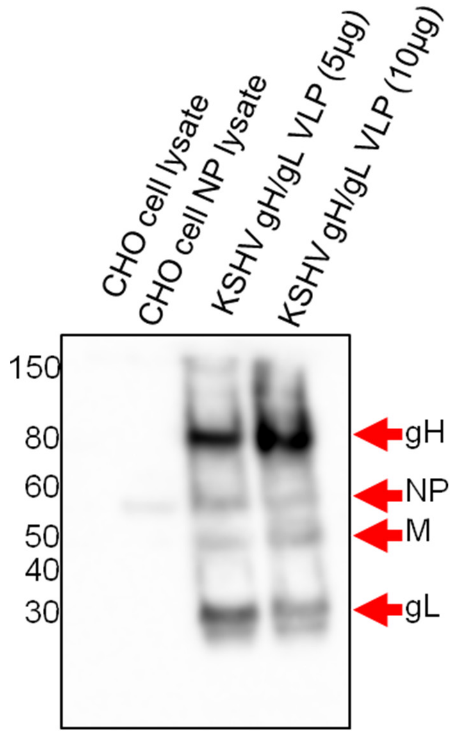

Supplementary Figure 2: Immunoblot analysis of KSHV gH/gL VLPs. CHO cells was co-transfected with pCAGGS-gH-F, gL-HN, -NDV NP, and -M vectors. Supernatants from transfected cells were harvested daily (24-96 h). VLPs in the supernatant were concentrated by centrifugation and purified using sucrose density gradients as described in Materials and Methods. Lysates from CHO cells (lane 1), CHO cells transfected with pCAGGS-NP alone (lane 2), or 5 and 10 µg purified VLPs (lanes 3-4) were lysed using RIPA buffer and boiled in non-reducing SDS-Laemmli buffer, followed by immunoblot. Serum from mice immunized six times with gH/gL VLPs for monoclonal antibody production (data not shown) detected KSHV gH, gL, NDV NP and M proteins in VLPs and NP alone in CHO cell NP lysate, as indicated, but not in CHO cells (negative control).

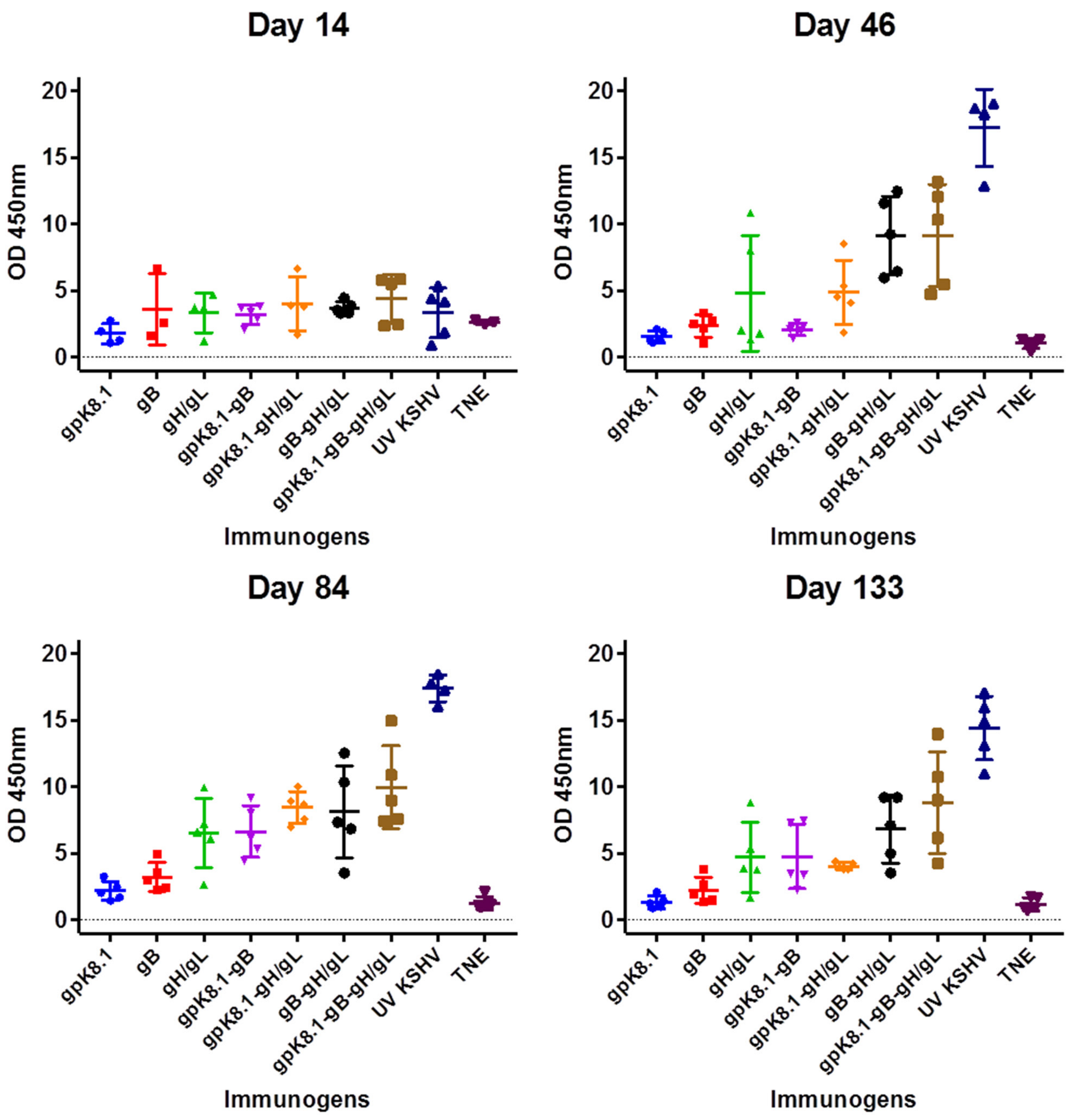

Supplementary Figure 3: Kinetics of specific anti-KSHV antibody responses in BALB/c mice. Antibody titers in sera from immunized BALB/c mice were determined using ELISA on Days 14, 46, 84, and 133. The absorbance resulting from serum antibody binding to plates coated with lysate from lytically induced KSHV-eGFP-expressing iSLK.219 cells is shown for individual mice from each vaccination group. Absorbance data are shown as the mean ± the SEM for five mice. Data points represent individual mice. KSHV VLPs induced increasing titers of anti-KSHV specific IgG antibodies after each immunization for each individual mouse.
